# Scaling-Up Access to Antiretroviral Therapy for Children: A Cohort Study Evaluating Care and Treatment at Mobile and Hospital-Affiliated HIV Clinics in Rural Zambia

**DOI:** 10.1371/journal.pone.0104884

**Published:** 2014-08-14

**Authors:** Janneke H. van Dijk, William J. Moss, Francis Hamangaba, Bornface Munsanje, Catherine G. Sutcliffe

**Affiliations:** 1 Macha Research Trust, Macha Hospital, Choma, Zambia; 2 Department of Immunology and Infectious Diseases, Erasmus University Rotterdam, the Netherlands; 3 Department of Epidemiology, Bloomberg School of Public Health, Johns Hopkins University, Baltimore, Maryland, United States of America; University of Missouri-Kansas City, United States of America

## Abstract

**Background:**

Travel time and distance are barriers to care for HIV-infected children in rural sub-Saharan Africa. Decentralization of care is one strategy to scale-up access to antiretroviral therapy (ART), but few programs have been evaluated. We compared outcomes for children receiving care in mobile and hospital-affiliated HIV clinics in rural Zambia.

**Methods:**

Outcomes were measured within an ongoing cohort study of HIV-infected children seeking care at Macha Hospital, Zambia from 2007 to 2012. Children in the outreach clinic group received care from the Macha HIV clinic and transferred to one of three outreach clinics. Children in the hospital-affiliated clinic group received care at Macha HIV clinic and reported Macha Hospital as the nearest healthcare facility.

**Results:**

Seventy-seven children transferred to the outreach clinics and were included in the analysis. Travel time to the outreach clinics was significantly shorter and fewer caretakers used public transportation, resulting in lower transportation costs and fewer obstacles accessing the clinic. Some caretakers and health care providers reported inferior quality of service provision at the outreach clinics. Sixty-eight children received ART at the outreach clinics and were compared to 41 children in the hospital-affiliated clinic group. At ART initiation, median age, weight-for-age z-scores (WAZ) and CD4^+^ T-cell percentages were similar for children in the hospital-affiliated and outreach clinic groups. Children in both groups experienced similar increases in WAZ and CD4^+^ T-cell percentages.

**Conclusions:**

HIV care and treatment can be effectively delivered to HIV-infected children at rural health centers through mobile ART teams, removing potential barriers to uptake and retention. Outreach teams should be supported to increase access to HIV care and treatment in rural areas.

## Introduction

In 2012, an estimated 230,000 children were newly infected with the human immunodeficiency virus (HIV) in sub-Saharan Africa, bringing the total number of children living with HIV in the region to 2.9 million [1]. Over the last decade, great progress has been made in providing these children access to lifesaving treatment. The number of children receiving antiretroviral therapy (ART) in sub-Saharan Africa increased from 85,000 in 2006 to 551,065 in 2012 [1], [2]. However, progress must continue so all eligible HIV-infected children have access to treatment as only 32% of children in need currently receive ART [1].

In much of sub-Saharan Africa, provision of free ART services has created demands on the health system that are not sustainable given current levels of infrastructure and human resources. This increased demand has necessitated a shift away from a medical model of care, primarily used in resource-rich countries and relying on highly trained medical personnel in specialized healthcare facilities to provide individualized HIV care and treatment, to a public health model that delivers treatment to more individuals [3]. The public health model relies on decentralization of HIV services to increase access, particularly in rural areas, task-shifting of activities to overcome the dearth of healthcare personnel [4], and standardized and simplified regimens and care packages to facilitate administration of care and treatment to large numbers of patients [5].

Different strategies for decentralizing HIV services have been developed in sub-Saharan African countries that are adapted to local conditions, influenced by varying roles of donor support, and incorporate lessons learned over the past decade. Many programs have implemented services at primary health centers, with care and treatment administered by general practitioners, clinical officers or nurses [6]–[20]. Several of these programs are assisted by mobile teams from hospital programs to support ART provision in primary health centers that do not have the full complement of facilities and human resources to provide comprehensive HIV services. Others have implemented home-based [21]–[23] and community-based [24] programs, with care and medication delivered by trained volunteers or field officers. By delivering care and treatment closer to home, these programs have succeeded in removing the main structural barriers, including transportation and distance to the clinic, to initiating and sustaining care [25]. Evaluations of these programs, many of which have been conducted among adults, have found improved retention and lower loss to follow-up compared with centralized, hospital-based care [6]–[9], [17], [20].

While decentralization has succeeded in increasing access to care for large numbers of patients, particularly in rural areas, one concern is that transitioning from specialized healthcare facilities and trained healthcare personnel may compromise the quality of care. Published evaluations of treatment outcomes in decentralized programs have largely been favorable and several, but not all [6], [16], programs found similar or better survival and clinical and virologic outcomes compared with hospital-based care [7]–[9], [17], [20]–[22]. However, additional evaluations are needed of decentralized programs, particularly for children, to ensure optimal care and the full benefits of treatment. In rural southern Zambia, a mobile ART program for children was evaluated and treatment outcomes were compared among HIV-infected children receiving care in mobile and hospital-affiliated HIV clinics.

## Methods

### Ethics statement

The study was approved by the Ministry of Health of the Government of Zambia, the University of Zambia Biomedical Research Ethics Committee and the Institutional Review Board of the Johns Hopkins Bloomberg School of Public Health. Caretakers provided written informed consent and children 8–16 years of age provided assent to participate in the study.

### ART provision in Zambia

In 2004, the Government of Zambia initiated public sector ART programs, with roll-out beginning in primary care clinics in the Lusaka Urban District [26] and subsequently implemented throughout the country. The program provides ART and basic laboratory tests, including CD4^+^ T-cell counts, free of charge. The number of ART sites increased from four in 2004 to 454 in 2010 [27].

While 61% of the Zambian population resides in rural areas [28], ART services are primarily offered in urban areas and in district level hospitals where the infrastructure and human and technical resources are greatest. To reach those affected by HIV in rural areas and increase access to HIV services, the Zambian Ministry of Health introduced the national Mobile ART Services program in 2007 [29]. Under this program, mobile ART teams of medical professionals were created at district hospitals. Rural health centers (RHC) in the catchment area were selected as designated ART outreach sites to be visited every two weeks by the mobile ART teams. Rural health center staff provide reproductive, maternal and child health care, treatment for tuberculosis, HIV testing, and other basic services, but generally do not have the training or capacity to provide ART. The mobile ART team assists staff at the ART outreach site in providing ART services, builds capacity and coordinates laboratory services. This program has contributed to the decentralization of ART services to the primary health care level to maximize limited resources and reach the greatest number of people in need.

### Study setting, clinical care and referral procedures

This study was conducted at the rural HIV clinic at Macha Hospital in Southern Province, Zambia. The study setting and population have been described in detail elsewhere [30], [31]. In brief, Macha Hospital serves as a referral hospital for at least 13 rural health centers, providing services for patients within an 80 km radius. The catchment area of Macha Hospital is populated by traditional villagers living in small, scattered homesteads, characteristic of much of rural sub-Saharan Africa, with an estimated population size of over 150,000 persons. The HIV clinic has provided care to more than 8500 HIV-infected adults and children since 2005. HIV care services, including antiretroviral treatment, are provided through the Government of Zambia’s antiretroviral treatment program, with support from the President’s Emergency Plan for AIDS Relief. The clinic provides care and treatment free of charge by physicians, clinical officers and nurses.

Children diagnosed with HIV infection are determined to be eligible for ART according to guidelines established by the Ministry of Health [32]. Children eligible for ART must undergo counseling to ensure the family is prepared to initiate ART. Upon initiation, children are seen every two weeks for the first month and every month for the following two months. Thereafter, the child is seen at three-monthly intervals if adherence and clinical response are good. Standard ART regimens consist of zidovudine, stavudine or abacavir plus lamivudine, and nevirapine or efavirenz.

Due to increasing patient numbers and considerable travel distance to access HIV services [30], the HIV clinic began a mobile ART program in 2007. Three rural health centers were selected as outreach clinics based on distance and size of the catchment populations, and were located 13 km (Mapanza RHC in Choma District), 21 km (Chilala RHC in Kalomo District) and 46 km (Moobola RHC in Namwala District) from the hospital. In 2010, Chilala RHC began providing ART services independently and children who were seen in the outreach clinic were officially transferred from the Macha HIV clinic. The selected outreach clinics are staffed with an average of one clinical officer and a minimum of two nurses.

The outreach team from the Macha Hospital clinic consists of at least one clinical officer or licentiate, nurse, pharmacy dispenser, laboratory assistant, counselor and data entry clerk. Medications, medical consumables and transportation are provided by the hospital. As laboratory testing cannot be performed at the outreach clinics, blood samples are collected and transported from the outreach clinic to the Macha Hospital laboratory, and results are returned during the next outreach visit. Clinically stable patients with good adherence are provided with a 3-month supply of medication.

Children are eligible for referral to the outreach clinic for care and treatment if they have been stable on ART for at least three months, demonstrated good adherence, have no opportunistic infections, and their caregiver requested to receive care closer to home. Children not yet eligible for ART and in stable condition can also be referred to the outreach clinic with the understanding that referral back to the hospital clinic might be needed once the child becomes eligible for ART or their condition worsens. Few children start ART at an outreach clinic.

### Study procedures

Beginning in September 2007, HIV-infected children younger than 16 years of age and registered at the HIV clinic at Macha Hospital were eligible for enrollment into an observational cohort study. This report describes a subset of these children receiving care between September 2007 and March 2012.

Children were evaluated at study visits approximately every three months. At each visit, a structured questionnaire was administered to the caregiver to collect information on socio-demographics, household characteristics, and medical and treatment history. The child was examined to measure height and weight, and a blood specimen was obtained to measure CD4^+^ T-cell counts and percentages (Guava Easy CD4 system; Guava Technologics, Inc., Hayward, CA) as part of clinical care. Plasma levels of HIV RNA were quantified by reverse transcriptase polymerase chain reaction assay (Amplicor HIV-1 Monitor v. 1.5, Roche Molecular Systems; lower limit of detection of 400 copies/mL, upper limit of detection 750,000 copies/mL) as part of the study. Due to financial constraints, viral load testing was not performed at all study visits; samples for viral load testing were selected every three months during the first year of ART and every six months thereafter. Adherence was assessed at every visit by pill counts and syrup volume measurements. For children who missed study visits, home visits were attempted to ascertain their status.

Upon implementation of the mobile ART program, some study children were transferred to outreach clinics and a study assistant was added to the outreach team to continue study procedures at outreach visits. A questionnaire was administered to the caretaker at one outreach clinic visit to obtain information on health care delivery system related factors, including access to care and perceived quality of care at the outreach clinic.

### Statistical analysis

Two analyses were conducted. The first analysis compared modes of transportation and travel time before and after transfer to the outreach clinic and assessed the perceived quality of care at the outreach clinics. HIV-infected children who transferred to the outreach clinic and whose caregiver completed a questionnaire both at study entry and upon transfer to the outreach clinic were eligible to be included in this analysis.

The second analysis compared treatment outcomes between children receiving ART who transferred to the outreach clinics and children receiving ART at the Macha HIV clinic who reported Macha Hospital as their hospital-affiliated rural health center. This comparison group was selected as these children live in the vicinity of the Macha HIV clinic and would not have been transferred to an outreach clinic, thereby removing potential confounding by distance to the clinic which is known to impact clinical outcomes. All children were required to have initiated ART before September 1, 2011 and have at least one study visit after ART initiation. Children in the outreach group were required to have transferred to an outreach clinic before September 1, 2011 and have at least one study visit after transfer.

Children in both groups remained in the analysis until the first of death, transfer, loss to follow-up, or administrative censoring on March 1, 2012. Transfer in this context was defined as transfer of care to a clinic other than one of the outreach clinics. Children attending Chilala Clinic when it became independent were censored on their last study visit. Loss to follow-up was defined as failure to attend a study visit for at least six months prior to March 1, 2012.

Descriptive statistics were used to compare the hospital-affiliated clinic group and outreach groups on characteristics at study entry and at ART initiation. A measure of socio-economic status (SES) was calculated based on the Demographic and Health Survey SES scale used in Zambia [33], with scores ranging from 0 to 24. SES percentiles were based on the predetermined cutoffs (<25^th^ = 0–6; 26–50^th^ = 7–12; 51–75^th^ = 13–18; >75^th^ = 19–24). Weight-for-age z-scores (WAZ) among children younger than 10 years of age were calculated based on the WHO growth standards [34], and children with z-scores below −2 were defined as underweight. Severe immunodeficiency was defined by CD4^+^ T-cell percentage according to the WHO 2006 treatment guidelines [35]. If laboratory tests were not available from the visit at which ART was initiated, results within three months prior to the date of initiation were used.

Immunologic, clinical and virologic treatment outcomes were assessed, including CD4^+^ T-cell percentage, WAZ and viral suppression. For CD4^+^ T-cell percentage and WAZ, children were included if they had at least one measure available after ART initiation. Both outcomes were evaluated using linear regression with generalized estimating equations (GEE) with robust variance estimation to account for repeated measures per child. Models for each outcome compared: 1) pre-transfer visits in the outreach group to all visits in the hospital-affiliated clinic group; 2) post-transfer visits in the outreach group to all visits in the hospital-affiliated clinic group; and 3) changes between last pre-transfer visit and the 6-month post-transfer visit in the outreach group to changes between visits at a similar interval (6 months) in the hospital-affiliated clinic group. Models included the duration of ART and other covariates found to differ (p<0.10) between children in the outreach and hospital-affiliated clinic groups and known to be causally associated with each outcome. Trajectories of each outcome were explored using linear mixed effects models with random intercept, exchangeable correlation structure and robust standard error estimation. As neither outcome was linear over time, a spline term was added at 7.5 months, the upper window around the 6-month measure. The primary exposure in the models was clinic (hospital-affiliated vs. outreach clinic), which was treated as a time-varying covariate. Interactions between clinic and time were included in the models to determine whether trajectories of the outcomes differed between children in the hospital-affiliated and outreach clinics.

For virologic outcomes, the proportion of children with viral suppression, defined as a HIV viral load below the limit of detection (400 copies/mL), was calculated for each visit after ART initiation. The proportion of children with viral suppression at each visit was compared between children in the hospital-affiliated and outreach clinic groups using chi-square tests. Unadjusted and adjusted logistic regression models with GEE were used to compare the odds of detectable viral load between children in the outreach and hospital-affiliated clinics (treated as a time-varying covariate) during follow-up. Detectable viral load was assessed at three thresholds: >400, >1000 and >10,000 copies/mL.

Adherence during follow-up was also assessed. Caregivers were instructed to bring all unused medications to each clinic visit and adherence was measured by pill count or measurement of liquids for each drug prescribed. Adherence measures were capped at 100%. For children taking individual drugs, the adherence percentage of the drug to which the patient was least adherent was used. Optimal adherence was defined as taking more than 95% of drugs prescribed. The proportions of children with optimal adherence at each visit and at all visits were compared between children in the hospital-affiliated and outreach clinics using chi-square tests. Unadjusted and adjusted logistic regression models with GEE were used to compare the odds of optimal adherence between children in the outreach and hospital-affiliated clinics (treated as a time-varying covariate) during follow-up.

All analyses were conducted using SAS for Windows version 9.1 (SAS Institute Inc., Cary, NC) and Stata, version 9 (StataCorp LP, College Station, TX).

## Results

### Comparison of HIV-infected children receiving care at the outreach clinics before and after transfer

**Figure 1:**
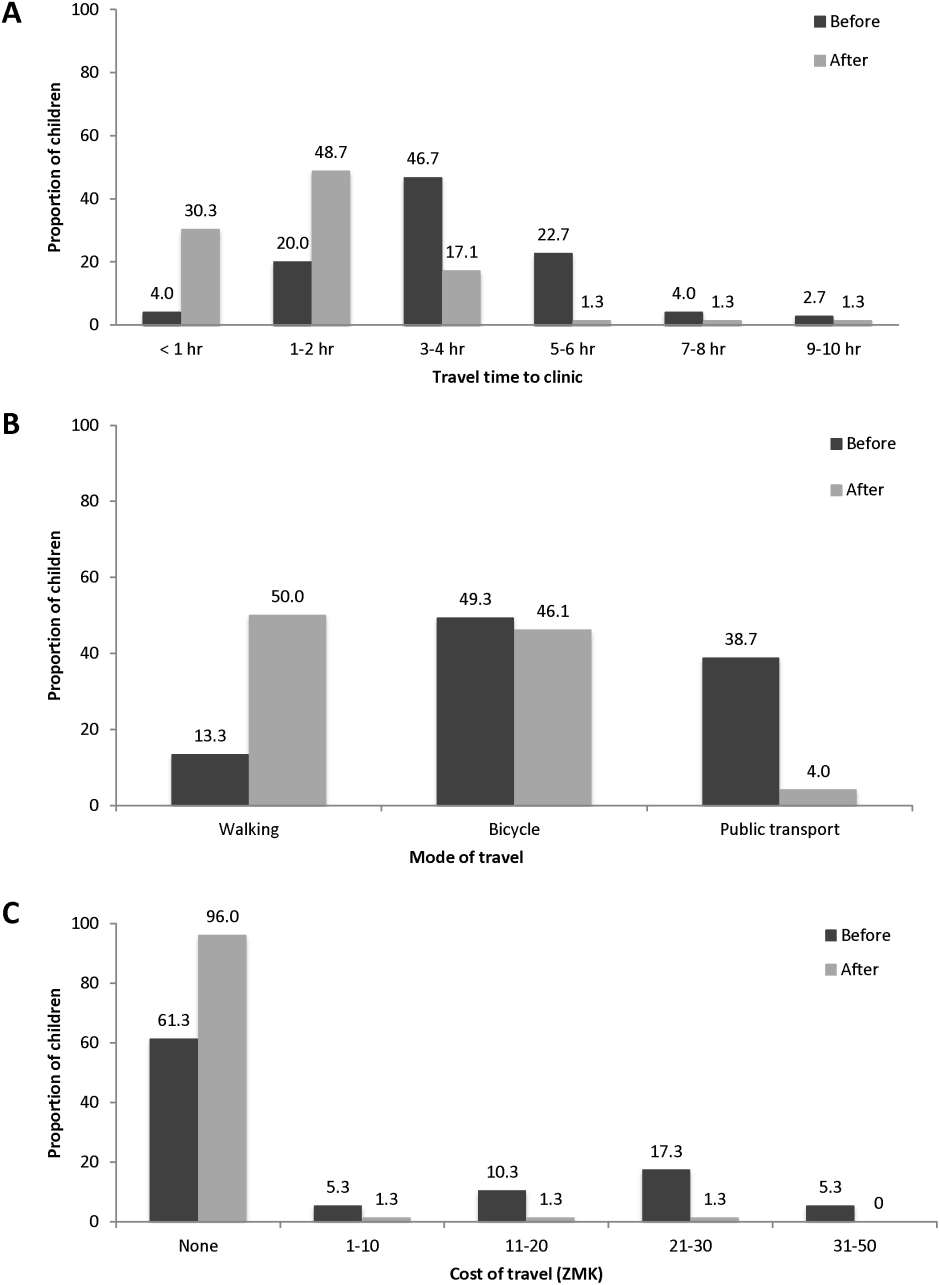
Travel time (A), mode (B), and cost (C) before and after transfer to outreach clinics. Note: participants were able to report more than one mode of travel to the clinic.

A total of 111 HIV-infected children were referred to one of the outreach clinics before September 1, 2011, and caretakers of 77 (69%) children completed the outreach questionnaire. The median age of the children at study entry was 3.4 years (IQR: 1.7, 7.7) and 47% were male. Almost all caretakers (99%) reported that it was easier to get to the outreach clinic compared to Macha Hospital because they did not have to travel as far (100%), had lower travel costs (56%), and transportation to the outreach clinic was easier (67%). After transfer to the outreach clinic, travel time was significantly shorter (p<0.0001). The proportion of children travelling more than five hours to get to the clinic decreased from 29% to 4% (figure 1). The proportion of children requiring public transport, as opposed to walking or using a bicycle, decreased from 39% to 4% (p<0.0001). Consequently, caretakers reported lower transportation costs and had fewer difficulties in finding transportation after transferring to the outreach clinic (91% vs. 21%; p<0.0001). The proportion of caretakers reporting no costs associated with travel increased from 61% to 96% (p<0.0001).

The majority of caretakers (83%) reported the overall quality of care at the outreach clinics to be the same as the hospital. When asked about specific components of care, however, many reported differences at the outreach clinics. Most caretakers reported the waiting time to be shorter (85%), but some reported the counseling services (34%) and physical examination (26%) in the outreach clinics to be of lower quality than at the hospital clinic.

### Comparison of HIV-infected children receiving ART at the hospital-affiliated and outreach clinics

#### Characteristics of the population at study entry and ART initiation

Forty-one children in the hospital-affiliated clinic group and 68 children in the outreach clinic group received ART during the study period, of whom 34 and 48, respectively, initiated ART during the study period. Children in the outreach clinic group were similar to the hospital-affiliated clinic group at study entry, except they were more likely to be younger (median age: 2.9 vs. 4.9 years; p = 0.07) and have both parents alive (75% vs. 54%; p = 0.03) (Table 1). At ART initiation, children in the outreach clinic group were more likely to be younger (median age: 2.9 vs. 5.9 years; p = 0.03) and to have a lower WAZ (median WAZ: −2.3 vs. −1.7; p = 0.07) (Table 1).

**Table 1:** Characteristics of HIV-infected children receiving ART at the hospital-affiliated and outreach clinics.

	Hospital-affiliated Clinic (n = 41)	Outreach Clinics (n = 68)	p-value
*At entry*	n (%)	n (%)	
Male	19 (46.3)	37 (54.4)	0.41
Median age in years (IQR)	4.9 (2.2, 9.4)	2.9 (1.6, 7.5)	0.07
Age (years)			0.70
<1	3 (7.3)	7 (10.3)	
1–1.9	7 (17.1)	16 (23.5)	
2–4.9	11 (26.8)	19 (27.9)	
≥5	20 (48.8)	26 (38.2)	
Status of parents			0.03
Both alive	22 (53.7)	51 (75.0)	
One parent alive	15 (36.6)	10 (14.7)	
Both parents died	4 (9.8)	7 (10.3)	
Other household member receiving ART	21 (52.5)	43 (64.2)	0.23
SES quartile			0.21
1^st^ (lowest)	28 (68.3)	41 (60.3)	
2^nd^	10 (24.4)	26 (38.2)	
3^rd^	2 (4.9)	1 (1.5)	
4^th^ (highest)	1 (2.4)	0 (0.0)	
Education of primary caregiver^a^			0.20
None	0 (0.0)	2 (3.1)	
Primary	19 (55.9)	43 (67.2)	
Secondary	14 (41.2)	19 (29.7)	
College/technical training	1 (2.9)	0 (0.0)	
Travel time (hours)^b^			0.53
<1	19 (46.3)	15 (31.9)	
1–2	15 (36.6)	21 (44.7)	
3–4	5 (12.2)	9 (19.2)	
≥5	2 (4.9)	2 (4.2)	
***At ART initiation***			
Median age in years (IQR)	5.9 (2.4, 10.4)	2.9 (1.7, 7.3)	0.03
Age (years)			0.15
<1	3 (7.3)	6 (8.8)	
1–1.9	4 (9.8)	17 (25.0)	
2–4.9	11 (26.8)	20 (29.4)	
≥5	23 (56.1)	25 (36.8)	
Median WAZ (IQR)	−1.7 (−2.5, −0.7)	−2.3 (−3.6, −1.3)	0.07
Underweight	12 (41.4)	34 (58.6)	0.13
Median CD4% (IQR)	14.4 (11.0, 19.5)	14.2 (10.5, 18.9)	0.91
Severe immunosuppression	23 (62.2)	36 (65.5)	0.75
ART regimen			0.31
AZT/3TC/EFV	10 (25.0)	15 (22.4)	
AZT/3TC/NVP	3 (7.5)	15 (22.4)	
D4T/3TC/EFV	16 (40.0)	24 (35.8)	
D4T/3TC/NVP	16 (40.0)	24 (35.8)	
Other	3 (7.5)	2 (3.0)	

3TC: lamivudine; ART: Antiretroviral therapy; AZT: zidovudine; D4T: stavudine; EFV: efavirenz; IQR: interquartile range; NVP: nevirapine; WAZ: weight-for-age z-score;

a among respondents who were primary caregivers, n = 98.

b after transfer to the outreach clinic among the outreach clinic group.

#### Clinical, immunological and virological responses to treatment

Children in the outreach and hospital-affiliated clinic groups were followed for a median of 32.3 (IQR: 22.3, 38.8) and 33.5 (IQR: 23.1, 42.6) months in the study while receiving ART (p = 0.50). Among children in the outreach clinic group, the median time between study enrolment and transfer to the outreach clinic was 9.1 months (IQR: 3.9, 14.4), and children received care at the outreach clinic for a median of 23.8 months (IQR: 12.2, 32.4) during the study. Two children started ART at the outreach clinic.

**Figure 2:**
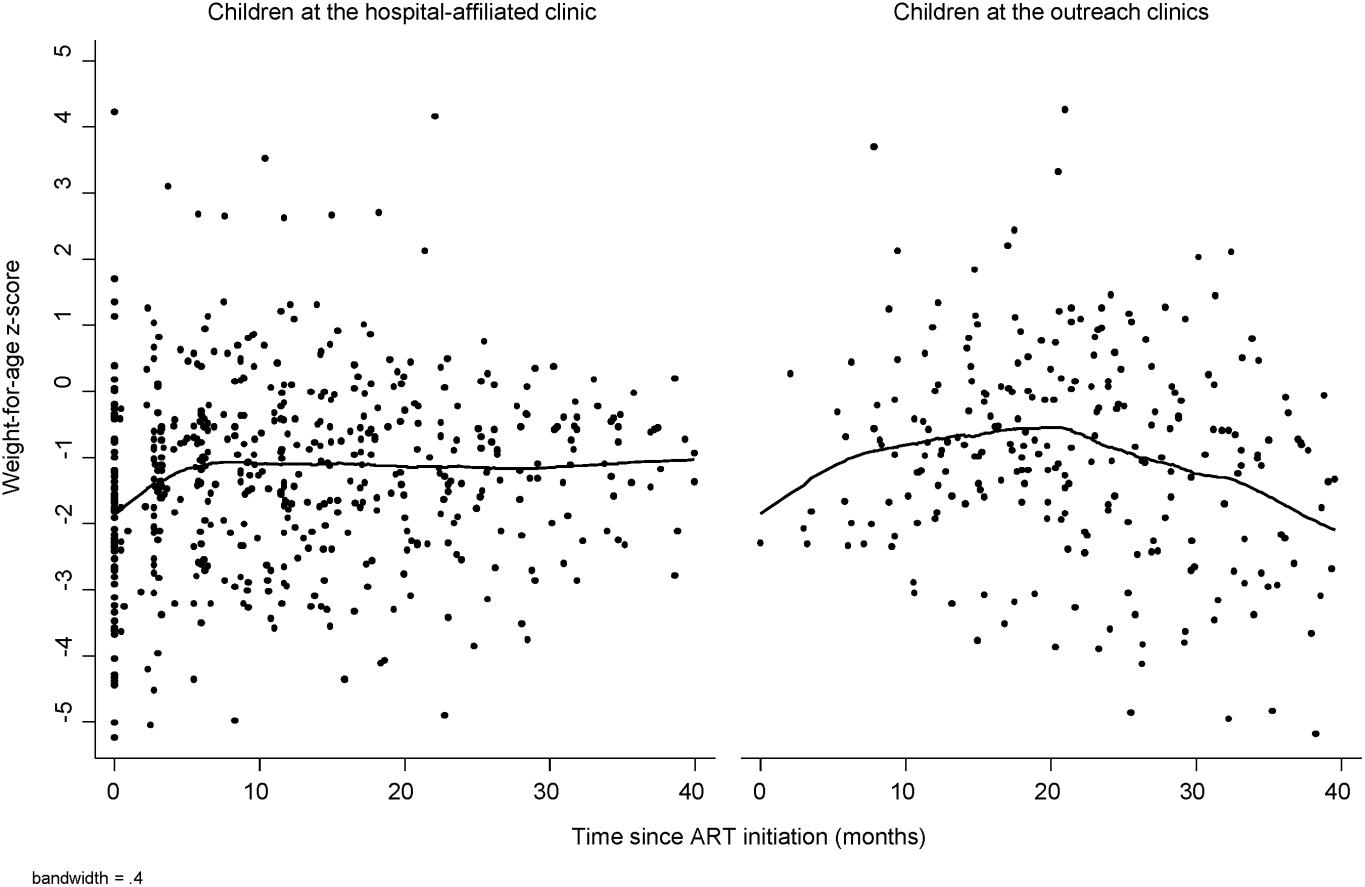
Lowess graph of weight-for-age z-score by time since ART initiation for HIV-infected children at the hospital-affiliated and outreach clinics. Note: Care at outreach clinics was treated as a time-varying covariate.

**Figure 3:**
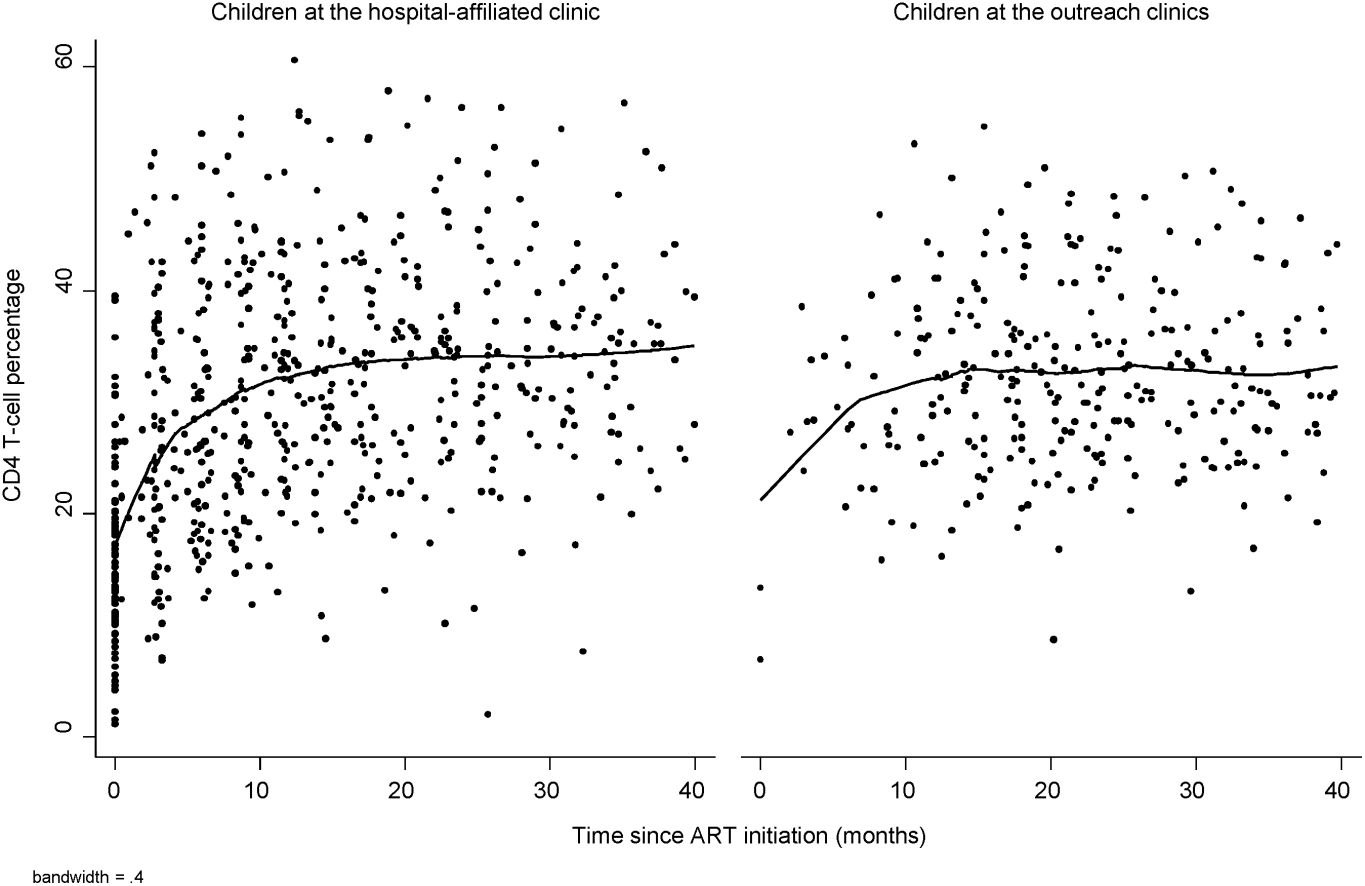
Lowess graph of CD4^+^ T-cell percentage by time since ART initiation for HIV-infected children at hospital-affiliated and outreach clinics. Note: Care at outreach clinics was treated as a time-varying covariate.

Children in both groups responded well to ART (Figures 2 and 3). Mean WAZ did not differ between the outreach and hospital-affiliated clinic groups either before (difference: −0.2; 95% confidence interval [CI]: −0.7, 0.3; p = 0.48) or after transfer (difference: 0.01; 95% CI: −0.5, 0.5; p = 0.96), after adjusting for month on ART and age, underweight, and severe immunosuppression at ART initiation (Table 2). Changes in WAZ among children in the outreach group between the last visit at the hospital-affiliated clinic and 6 months after transfer also did not differ significantly from changes in WAZ among children in the hospital-affiliated clinic group over the same time interval (Table 2). These findings are supported by longitudinal models comparing trajectories between the two groups (Table S1).

**Table 2:** Differences in mean weight-for-age z-scores and CD4^+^ T-cell percentage between children receiving ART at the hospital-affiliated and outreach clinics.

	Comparison of the outreach clinic group to the hospital-affiliated clinic group	Estimate (95% CI)^a^^,^ b	p-value^a^
**WAZ**			
1	**Outreach**: all visits before transfer		
	**Hospital-affiliated**: all visits	−0.2 (−0.7, 0.3)	0.48
2	**Outreach**: all visits after transfer		
	**Hospital-affiliated**: all visits	0.01 (−0.5, 0.5)	0.96
3	**Outreach**: Difference between visit 6 month after transfer and last visit before transfer		
	**Hospital-affiliated**: Difference between visits at 6 months intervals	0.4 (−0.07, 0.8)	0.10
**CD4%**			
1	**Outreach**: all visits before transfer		
	**Hospital-affiliated**: all visits	−1.0 (−4.2, 2.2)	0.55
2	**Outreach**: all visits after transfer		
	**Hospital-affiliated**: all visits	−3.3 (−6.8, 0.2)	0.06
3	**Outreach**: Difference between visit 6 month after transfer and last visit before transfer		
	**Hospital-affiliated**: Difference between visits at 6 month intervals	−1.4 (−4.5, 1.7)	0.37

CI: Confidence interval; WAZ: weight-for-age z-score.

a Estimates and p-values for comparisons from linear regression with GEE to account for repeated measures per child.

b Adjusted for month on ART, age at ART initiation, underweight at ART initiation and severe immunosuppression at ART initiation.

Mean CD4^+^ T-cell percentage did not differ between the outreach and hospital-affiliated clinic groups before transfer (difference: −1.0; 95% CI: −4.2, 2.2; p = 0.55) and was non-significantly lower in the outreach group after transfer (difference: −3.3; 95% CI: −6.8, 0.2; p = 0.06), after adjusting for month on ART and age, underweight, and severe immunosuppression at ART initiation (Table 2). Changes in CD4^+^ T-cell percentage among children in the outreach group between the last visit at the hospital-affiliated clinic and 6 months after transfer did not differ significantly from changes in CD4^+^ T-cell percentage among children in the hospital-affiliated clinic group over the same time interval (Table 2). These findings are supported by longitudinal models comparing trajectories between the two groups (Table S1).

Viral suppression was assessed up to three years after ART initiation. The proportion of children with undetectable viral load was lower among children receiving care at the outreach clinics at each time point, although samples sizes were small and few differences were statistically significant (Table 3). Considering all measures during follow-up (n = 473), viral load measures from children in the outreach clinics were significantly more likely to be detectable at >400 copies/mL (17% vs. 8%; p = 0.002), >1000 copies/mL (16% vs. 7%; p = 0.002) and >10,000 copies/mL (10% vs. 3%; p = 0.001) than from children in the hospital-affiliated clinic. In a crude model, the odds of viral load >400 copies/mL (odds ratio [OR]: 1.62; 95% CI: 0.75, 3.50), viral load >1000 copies/mL (OR: 1.59; 95% CI: 0.67, 3.79), and viral load >10,000 copies/mL (OR: 2.50; 95% CI: 0.87, 7.18) were higher for children in the outreach clinics compared to children in the hospital-affiliated clinic, although these results were not statistically significant. Adjusting for age and underweight at ART initiation and month of ART did not significantly affect the results (>400 copies/mL: OR: 1.52, 95%: 0.56, 4.10; >1000 copies/mL: OR: 1.85, 95% CI: 0.72, 4.76; >10,000 copies/mL: 5.14, 95% CI: 1.50, 17.61).

**Table 3:** Plasma HIV viral loads over time among children receiving ART at hospital-affiliated and outreach clinics.

	Hospital-affiliated Clinic		Outreach Clinics^a^		
Month on ART	N	% undetectable VL	N	% undetectable VL	p-value
3	44	90.9	3	100.0	0.59
6	61	90.2	6	100.0	0.42
9	34	94.1	8	50.0	0.001
12	52	94.2	24	87.5	0.31
18	28	100.0	29	82.8	0.02
24	30	90.0	26	76.9	0.18
30	12	91.7	16	81.3	0.44
36	11	81.8	13	76.9	0.77

ART: antiretroviral therapy; VL: viral load.

a Outreach treated as a time-varying covariate.

#### Adherence

The proportion of children with optimal adherence (>95%) at each study visit tended to be lower for those receiving care at the outreach clinics compared to the hospital-affiliated clinic, although no significant differences were observed (Table S2). In a crude model, the odds of optimal adherence were non-significantly lower for children in the outreach clinics compared to the hospital-affiliated clinic (odds ratio [OR]: 0.71; 95% confidence interval [CI]: 0.45, 1.12). Adjusting for age and underweight at ART initiation and month of ART did not significantly impact the estimate (OR: 0.69; 95% CI: 0.41, 1.15). To further investigate adherence, all visits after ART initiation (combining visits at the hospital-affiliated and outreach clinics for children in the outreach clinic group) were considered. Children receiving care at the outreach clinic had poorer adherence, with the median percentage of visits with optimal adherence (69.2% vs. 79.3%; p = 0.01) and the proportion of children with optimal adherence at all study visits (24.6% vs. 32.5%; p = 0.35) lower in the outreach compared to the hospital-affiliated clinic group. When adherence among children in the outreach clinic group was stratified by location, no significant difference in adherence was found before and after transfer to the outreach clinics. The median percentage of visits with optimal adherence was 75% (IQR: 50, 100) before and 75% (IQR: 43, 100) after transfer (p = 0.81). The proportion of children with optimal adherence at all visits before and after transfer was 39% and 34% (p = 0.59), respectively.

#### Retention in care

At the end of the study, after a median of 34 months on treatment, 75% of children in the outreach clinic group and 95% of children in the hospital-affiliated clinic group were active in the program. Among children followed at the hospital-affiliated clinic, none died or were lost to follow-up and two children (4.9%) were transferred to other clinics. Among children followed at the outreach clinics, one died from drowning (1.5%) and none were lost to follow-up. The primary reason for departure from the program in the outreach clinic group was transfer: four children (5.9%) were transferred to other clinics and 12 children (17.6%) were transferred to Chilala Clinic when it became an independent ART clinic.

## Discussion

Decentralization from higher to lower level health facilities and task-shifting from higher to lower level care providers are essential if access to HIV care is to increase in resource-limited countries in the face of rising patient numbers [7]. Bringing care closer to patients can provide benefits beyond simply being able to treat more people. Reducing the travel distance and time required for patients to access clinics can improve retention in care, adherence to ART, and quality of life. In addition, increasing the number of healthcare facilities providing services can reduce the patient load at all facilities, thereby decreasing waiting times and increasing the amount of time that healthcare providers can spend with each patient. All of these factors have the potential to positively impact treatment outcomes.

Distance to the clinic and transportation were barriers to retention in care in rural southern Zambia and elsewhere [25], [30], with levels of attrition increasing with travel distance [36]. One multisite analysis in western, eastern and southern Africa found that the risk of attrition doubled if travel time to clinic exceeded 2 hours [25]. Distance to the clinic has also been found to be associated with an increased risk of virologic failure [31]. As HIV care is lifelong, making services more accessible and providing care in a familiar environment increases health-related quality of life and satisfaction with clinical services [37], [38], thus increasing the likelihood of retention in care. Several decentralized HIV treatment programs reported similar or better outcomes in HIV-infected adults [7]–[9], [21], [22], [38] compared with those treated at hospital-based HIV clinics. A recent study in five countries in sub-Saharan Africa also reported significantly lower rates of loss to follow-up (adjusted rate ratio: 0.55) and mortality (adjusted rate ratio: 0.66) among children cared for at primary health centers compared to secondary or tertiary health centers [17]. In this evaluation of mobile and hospital-affiliated HIV care for children in rural southern Zambia, transfer to the outreach clinic succeeded in significantly decreasing travel time and costs, barriers accessing the clinic, and reported waiting times at the clinic. Children receiving ART at the outreach clinics were able to achieve and maintain similar clinical and immunological responses to treatment compared to children receiving care at the hospital-affiliated clinic.

While there are many benefits to decentralization, challenges remain. Some studies have reported higher mortality among HIV-infected adults and children cared for at health centers compared to hospital clinics [6], and higher percentages of adults with detectable viral load among those followed in primary health clinics [16]. In this study, children in the outreach group tended to have lower levels of viral suppression. This may have been due to the lower observed adherence, which was observed while these children received care in the hospital-affiliated clinic and persisted after transfer to the outreach clinics. Reasons for lower adherence were not determined but may be due to several factors, including non-disclosure and disrupted routines (e.g., changes in address and school schedules) [39]. Adequate resources will need to be available at outreach clinics to monitor adherence and manage treatment failure. Discussions with the outreach team indicated that services could be improved at the outreach clinics with additional staff, particularly counselors, and space dedicated for clinical exams and counseling sessions. This was also reflected in perceptions by caregivers that these aspects of care were of lower quality. Involving community volunteers, including members of surrounding communities who are HIV-infected, peer educators or adherence support workers to improve adherence counseling [40], [41] may provide the necessary support for outreach teams to ensure that HIV-infected patients are adequately monitored.

There were several limitations that should be considered in interpreting the study results. First, this was an observational study in which children were transferred to the outreach clinics at various times after starting treatment at the request of the caregiver. Only children who responded well to treatment were eligible for transfer and therefore the outreach clinic group represented a select group of children. As a valid comparison group, children of caretakers who named Macha Hospital as their rural health center were selected to remove the effect of travel distance. Children in both groups were similar on many characteristics and measured differences were adjusted for in the analysis. However, unmeasured differences between the groups may have remained. Second, the sample size was small, particularly at longer follow-up times and for virologic outcomes. Lastly, this study was conducted at one hospital-affiliated clinic and three outreach clinics in a rural area in Zambia. The generalizability of the results to other settings may be limited.

## Conclusions

This is one of the few studies of HIV-infected children conducted in rural sub-Saharan Africa comparing treatment outcomes between different service delivery approaches. HIV care and treatment can be effectively delivered to HIV-infected children at rural health centers through mobile ART teams, removing potential barriers to uptake and retention. Outreach teams should be supported to continue to increase access to HIV services in rural areas and ensure that HIV-infected children receive optimal care and treatment.

## Supporting Information

Table S1 Crude and adjusted changes in weight-for-age z-scores and CD4^+^ T-cell percentages among children receiving ART at hospital-affiliated and outreach clinics.
(DOCX)

Table S2 Adherence over time among children receiving ART at the hospital-affiliated and outreach clinics.
(DOCX)
