# Correction: Scaling-Up Access to Antiretroviral Therapy for Children: A Cohort Study Evaluating Care and Treatment at Mobile and Hospital-Affiliated HIV Clinics in Rural Zambia

**DOI:** 10.1371/journal.pone.0112994

**Published:** 2014-11-04

**Authors:** The PLOS ONE Staff

There is an error in the funding statement. Please refer to the correct funding statement below.

This work was made possible by support from the President's Emergency Plan for AIDS Relief (PEPFAR) through Cooperative Agreement 5U2GPS001930-05 from the Department of Health and Human Services (DHHS)/Centers for Disease Control and Prevention (CDC), Global AIDS Program. The findings and conclusions included in its content are solely the responsibility of the author (s) and do not necessarily represent the official position of the Centers for Disease Control and Prevention/Agency for Toxic Substances and Disease Registry. Publication of this article was funded in part by the Open Access Promotion Fund of the Johns Hopkins University Libraries. This work was supported through technical assistance from the Johns Hopkins Center for AIDS Research (1P30AI094189). The funders had no role in study design, data collection and analysis, decision to publish, or preparation of the manuscript.

## References

[1] van Dijk JH, Moss WJ, Hamangaba F, Munsanje B, Sutcliffe CG (2014) Scaling-Up Access to Antiretroviral Therapy for Children: A Cohort Study Evaluating Care and Treatment at Mobile and Hospital-Affiliated HIV Clinics in Rural Zambia. PLoS ONE 9(8): e104884 doi:10.1371/journal.pone.0104884 25122213 10.1371/journal.pone.0104884 PMC4133342

