# Supplementary material for: Scaling-Up Access to Antiretroviral Therapy for Children: A Cohort Study Evaluating Care and Treatment at Mobile and Hospital-Affiliated HIV Clinics in Rural Zambia
Source: PLoS One. 2014 Aug 14;9(8):e104884. doi: 10.1371/journal.pone.0104884 (PMC4133342; doi:10.1371/journal.pone.0104884)
Supplement: Table S1 — Crude and adjusted changes in weight-for-age z-scores and CD4+ T-cell percentages among children receiving ART at hospital-affiliated and outreach clinics. (DOCX) [file pone.0104884.s001.docx]

**Table S1. Crude and adjusted changes in weight-for-age z-scores and CD4^+^ T-cell percentages among children receiving ART at hospital-affiliated and outreach clinics**

|  | **Crude^a^** | | | **Adjusted^a^** | | |
| --- | --- | --- | --- | --- | --- | --- |
|  | **Hospital-affiliated Clinic** | **Outreach Clinics** | **p-value** | **Hospital-affiliated Clinic** | **Outreach Clinics** | **p-value** |
| ***Weight-for-age z-score (WAZ)*** |  |  |  |  |  | ^b^ |
| WAZ at ART initiation (SE) | -1.97 (0.18) | -1.60 (0.54) | 0.50 | -1.70 (0.51) | -1.27 (0.74) | 0.44 |
| Increase in WAZ per month in first 6 months of ART (SE) | 0.11 (0.02) | 0.09 (0.07) | 0.78 | 0.11 (0.02) | 0.08 (0.08) | 0.70 |
| Increase in WAZ per month after 6 months of ART (SE) | -0.003 (0.007) | -0.002 (0.009) | 0.90 | -0.003 (0.007) | -0.003 (0.009) | 0.97 |
| ***CD4^+^ T-cell percentage (CD4%)*** |  |  |  |  |  | ^c^ |
| CD4^+^ T-cell percentage at ART initiation (SE) | 17.9 (1.00) | 23.7 (3.64) | 0.12 | 25.7 (3.62) | 28.8 (4.59) | 0.31 |
| Increase in CD4^+^ T-cell percentage per month in first 6 months of ART (SE) | 1.8 (0.12) | 1.1 (0.48) | 0.15 | 2.0 (0.13) | 1.4 (0.43) | 0.20 |
| Increase in CD4^+^ T-cell percentage per month after 6 months of ART (SE) | 0.07 (0.03) | 0.009 (0.05) | 0.16 | 0.07 (0.03) | 0.05 (0.05) | 0.66 |

ART: antiretroviral therapy; SE: standard error; WAZ: weight-for-age z-score

^a^ results from linear mixed effects model with random intercept, exchangeable correlation structure and robust standard error, with a spline at 7.5 months and interaction terms between clinic and time

^b^ adjusted for age at ART initiation

^c^ adjusted for age and WAZ at ART initiation

**Footnote:** Children in both groups experienced similar trajectories in WAZ and CD4+ T-cell percentage over time, with both measures increasing the first six months and then remaining stable. ***Interpretation of results for WAZ:*** In the first six months, mean WAZ, adjusted for age at ART initiation, increased 0.11 (SE: 0.02) per month among children receiving care at the hospital-affiliated clinic, and 0.08 (SE: 0.08) per month among children receiving care at the outreach clinics (p=0.70). After six months of ART, WAZ did not significantly change (mean increase per month: hospital-affiliated clinic group: -0.003; outreach clinic: -0.003; p=0.97). ***Interpretation of results for*** ***CD4^+^ T-cell percentage:*** In the first six months, mean CD4^+^ T-cell percentage, adjusted for age and WAZ at ART initiation, increased 1.97% (SE: 0.13) per month among children receiving care at the hospital-affiliated clinic, and 1.42% (SE: 0.43) per month among children receiving care at the outreach clinics. After six months of ART, CD4^+^ T-cell percentage did not significantly change (mean increase per month: hospital-affiliated clinic: 0.07%; outreach clinic: 0.05%; p=0.66).
