# Supplementary material for: Scaling-Up Access to Antiretroviral Therapy for Children: A Cohort Study Evaluating Care and Treatment at Mobile and Hospital-Affiliated HIV Clinics in Rural Zambia
Source: PLoS One. 2014 Aug 14;9(8):e104884. doi: 10.1371/journal.pone.0104884 (PMC4133342; doi:10.1371/journal.pone.0104884)
Supplement: Table S2 — Adherence over time among children receiving ART at the hospital-affiliated and outreach clinics. (DOCX) [file pone.0104884.s002.docx]

**Table S2: Adherence over time among children receiving ART at the hospital-affiliated and outreach clinics**

|  | **Hospital-affiliated Clinic** | | **Outreach Clinics** | |  |
| --- | --- | --- | --- | --- | --- |
| **Month on ART^a^** | **N** | **% with >95% adherence** | **N** | **% with >95% adherence** | **p-value** |
| 3 | 68 | 69.1 | 6 | 50.0 | 0.34 |
| 6 | 71 | 63.4 | 6 | 83.3 | 0.33 |
| 9 | 60 | 75.0 | 17 | 64.7 | 0.40 |
| 12 | 55 | 81.8 | 24 | 70.8 | 0.27 |
| 15 | 46 | 78.3 | 25 | 64.0 | 0.19 |
| 18 | 40 | 75.0 | 28 | 75.0 | 1.00 |
| 21 | 34 | 85.3 | 33 | 69.7 | 0.13 |
| 24 | 32 | 81.3 | 28 | 75.0 | 0.56 |
| 27 | 31 | 71.0 | 26 | 73.1 | 0.86 |
| 30 | 25 | 80.0 | 16 | 62.5 | 0.22 |
| 33 | 22 | 86.4 | 24 | 66.7 | 0.12 |
| 36 | 18 | 66.7 | 16 | 75.0 | 0.28 |
|  |  |  |  |  |  |
| Median % of visits with optimal adherence | 40 | 79.3 | 65 | 69.2 | 0.01 |
| % children with optimal adherence at all visits | 40 | 32.5 | 65 | 24.6 | 0.38 |

^a^ Outreach treated as a time-varying covariate
